# More Is Better: Recent Progress in Multi-Omics Data Integration Methods

**DOI:** 10.3389/fgene.2017.00084

**Published:** 2017-06-16

**Authors:** Sijia Huang, Kumardeep Chaudhary, Lana X. Garmire

**Affiliations:** ^1^Epidemiology Program, University of Hawaii Cancer CenterHonolulu, HI, United States; ^2^Molecular Biosciences and Bioengineering Graduate Program, University of Hawaii at ManoaHonolulu, HI, United States; ^3^Department of Obstetrics, Gynecology, and Women's Health, John A. Burns School of Medicine, University of Hawaii at ManoaHonolulu, HI, United States

**Keywords:** multi-omics, integration, prognosis, prediction, precision medicine, supervised learning, unsupervised learning

## Abstract

Multi-omics data integration is one of the major challenges in the era of precision medicine. Considerable work has been done with the advent of high-throughput studies, which have enabled the data access for downstream analyses. To improve the clinical outcome prediction, a gamut of software tools has been developed. This review outlines the progress done in the field of multi-omics integration and comprehensive tools developed so far in this field. Further, we discuss the integration methods to predict patient survival at the end of the review.

## Introduction

A new era of personalized medicine has arrived, which proposes an individualized health care model with tailored medical target treatment and management for each patient (Chin et al., 2011). Under this regime, not only clinical profiles of patients but also their molecular profiles are personally managed to drive for advanced treatment. Cancer studies that are focused on one-dimensional omics data have only provided limited information regarding the etiology of oncogenesis and tumor progression. To overcome this, tremendous efforts have been made to obtain multi-platform based genomic data from biospecimen.

The Cancer Genome Atlas (TCGA) is by far the largest endeavor in the USA to collect and analyze the tumor specimens from over 10,000 cancer patients (Weinstein et al., 2013). Measurements of these specimens include tissue exome sequencing, copy number variation (CNV), DNA methylation, gene expression, and microRNA (miRNA) expression, as well as some physiological and clinical data such as race, tumor stage, relapse, and treatment response. However, relative to the genomic data of different levels that are available to the public, the clinical information is more limited. A scale-up of TCGA is the International Cancer Genome Consortium (ICGC), which provides the information of genomic, transcriptomic and epigenomic abnormalities, and somatic mutations over 50 different cancer types (Hudson et al., 2010). These consortia have created unprecedented opportunities to reveal underlying oncogenic molecular signatures beneath phenotypes.

However, human genomes are complex and regulated at multiple levels, which can be manifested by various genomic assays mentioned above. While each of these assays offers a peek of the complex system, these events are rather interdependent (or interactive). Thus, when combining several different omics data to discover the coherent biological signatures, it is challenging to incorporate different biological layers of information to predict phenotypic outcomes (tumor/normal, early/late stage, survival, etc.). It is herein our goal to address the pressing and challenging issues for developing novel algorithms and theoretical methods for multi-omics data integration, in the hope to extract biologically meaningful information of clinical relevance.

The outline of this review is as follows. First, we will discuss the unsupervised data integration algorithms. Among them, we will highlight matrix factorization methods, Bayesian methods, and network-based methods. Next, we will review in-depth the supervised data integration methods, including network-based models, multiple kernel learning methods, and multi-step analysis based models. Subsequently, we will elaborate semi-supervised data integration methods. Finally, we will discuss the advancement of data integration methods for the aim of prognosis prediction and the biological insights underneath the data integration methods.

## Unsupervised data integration

**Figure 1:**
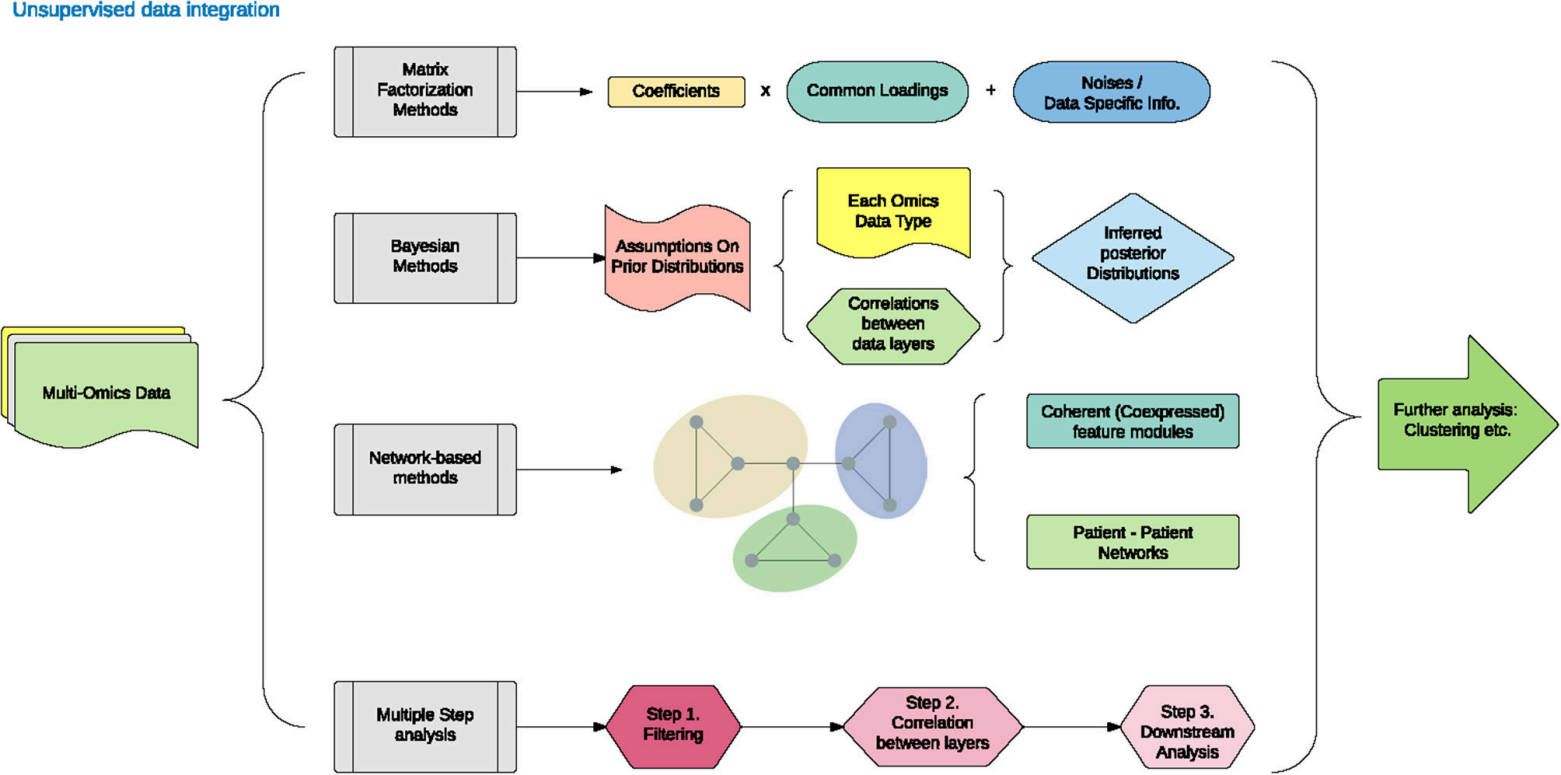
Unsupervised data integration methodology.

Unsupervised data integration refers to the cluster of methods that draw an inference from input datasets without labeled response variables. The different approaches under the umbrella of unsupervised data integration are presented in Figure 1 and Table 1. We have categorized them below into five areas: matrix factorization methods, Bayesian methods, network-based methods and multiple kernel learning, and multi-step analysis.

**Table 1:** Summary of data integration tools.

**Name**	**Category**	**Data type**	**Output**	**Stats method**	**FS method**	**References**
Joint NMF	Unsupervised	Multi-data	Subset of genes (modules)	Matrix factorization	NA	Zhang et al., 2011, 2012
iCluster	Unsupervised	EXP, CNV	Cluster	matrix factorization	L1 penalty	Shen et al., 2012
iCluster+	Unsupervised	Multi-data	Cluster	matrix factorization	L1 penalty	Mo et al., 2013
JIVE	Unsupervised	Multi-data	Shared factors and unique factors	Matrix factorization	L1 penalty	Lock et al., 2013
Joint Bayes Factor	Unsupervised	EXP, MET, CNV	Shared factors and unique factors	Matrix factorization	Student-t sparseness promoting prior	Ray et al., 2014
ssCCA	Unsupervised	Sequence data	Operational taxonomic unit and cluster	Canonical Correlation Analysis	L1 penalty	Chen et al., 2013
CCA sparse group	Unsupervised	Two types of data	Group of features with weights	Canonical Correlation Analysis	L1 penalty	Lin et al., 2013
sMBPLS	Unsupervised	Multi-data	Group of features as modules	Partial Least Squares	L1 penalty	Li et al., 2012
SNPLS	Unsupervised	EXP, drug response, gene network info.	Gene-drug co-module	Partial Least Squares	Network-based penalty	Chen and Zhang, 2016
MDI	Unsupervised	Multi-data	Cluster	Bayesian	NA	Kirk et al., 2012
Prob_GBM	Unsupervised	EXP, CNV, miRNA, SNP	Cluster	Bayesian	NA	Cho and Przytycka, 2013
PSDF	Unsupervised	EXP, CNV	Cluster	Bayesian	Binary indicator->likelihood of feature	Yuan et al., 2011
BCC	Unsupervised	EXP, MET, miRNA, proteomics	Cluster	Bayesian	NA	Lock and Dunson, 2013
CONEXIC	Unsupervised	EXP, CNV	Groups of genes associated with modulators	Bayesian	NA	Akavia et al., 2010
PARADIGM	Unsupervised	Multi-data	Gene score and significance in each pathway	pathway networks	NA	Vaske et al., 2010
SNF	Unsupervised	EXP, MET, miRNA	Cluster	similarity network fusion	NA	Wang et al., 2014
Lemon-Tree	Unsupervised	EXP, CNV/miRNA/ methyl (only one type)	Association network graphics	module network	NA	Bonnet et al., 2015
rMKL-LPP	Unsupervised	Multi-data	Cluster	Multiple kernel learning	Dimension reduction metric Locality Preserving Projections (LPP)	Speicher and Pfeifer, 2015
CNAmet	Unsupervised	EXP, MET, CNV	Scores and *p*-values of genes	Multi-step analysis	NA	Louhimo and Hautaniemi, 2011
iPAC	Unsupervised	EXP, CNV	Subset of genes	Multi-step analysis	Multiple filtering steps including common aberrant genes, in-cis correlation and in-trans functionality	Aure et al., 2013
ATHENA	Supervised	EXP, CNV, MET, miRNA	Final model with patient index	Grammatical Evolution Neural Networks (GENN)	Neural Networks	Kim et al., 2013
jActiveModules	Supervised	EXP, PPI, protein-DNA interactions	Subnetwork (network hotspots)	Network simulated annealing	NA	Ideker et al., 2002
Network propagation	Supervised	Gene expression, mutation, PPI	Propagated network relative to differential expression of gene	Network	NA	Ruffalo et al., 2015
SDP/SVM	Supervised	EXP, protein sequence, protein interactions, hydropathy profile	Linear classifier based on combination of kernels	SDP/SVM	Recommends CCA (canonical correlation analysis)	Lanckriet et al., 2004
FSMKL	Supervised	EXP, CNV, Clinic feature (ER status)	Linear classifier based on combination kernel	Multiple kernel learning	SimpleMKL (gradient descent method)	Seoane et al., 2014
iBAG	Supervised	Multi-data	Subset of genes	Multi-step analysis	Bayesian	Jennings et al., 2013
MCD	Supervised	MET, CNV, LoH	Subset of genes	Multi-step analysis	NA	Chari et al., 2010
Anduril	Supervised	EXP, MET, miRNA, exon, aCGH, SNP	Comprehensive report	Multi-step analysis	NA	Ovaska et al., 2010
GeneticInterPred	Semi-supervised	EXP, PPI, protein complex data	Genetic interaction labels	Graph integration	NA	You et al., 2010
Graph-based learning	Semi-supervised	EXP, CNV, MET, miRNA	Patient scores for classification purpose	Graph integration	NA	Kim et al., 2012
CoxPath	Survival-driven	EXP, CNV, MET, miRNA	Prognosis index for each patient	Multi-step analysis	L1 penalty	Mankoo et al., 2011
MKGI	Survival-driven	EXP, CNV, MET, miRNA	Final model with patient index	Grammatical Evolution Neural Networks (GENN)	Neural Networks	Kim et al., 2016

*FS Method, Feature Selection Method; EXP, Expression; CNV, Copy Number Variation; MET, DNA Methylation; SNP, Single Nucleotide Polymorphism; aCGH, Array Comparative Genomic Hybridization; PPI, Protein-Protein Interaction; LoH, Loss of Heterozygosity*.

### Matrix factorization methods

#### Joint Non-negative Matrix Factorization (NMF)

The most straightforward method for unsupervised data integration falls into the matrix factorization category, which focuses on the projection of variations among data sets onto dimension-reduced space (Lee and Seung, 2001). Zhang et al. proposed NMF framework for multi-omics data integration (Zhang et al., 2011, 2012). This method is based on decomposing a non-negative matrix into non-negative loadings and non-negative factors:

(1) $$\begin{array}{l} min||X-WH|{|}^{2},W\geq 0,H\geq 0 \end{array}$$

where *X* is the matrix of mRNA transcriptome, methylome, or other omics data that has M × N dimensions, *W* is the common factor for M × K dimension matrix and *H* is the K × N dimension coefficient matrix. Rather than simple correlation, the rationale is to project data onto common basis space, so that one can detect the coherent patterns among data, by examining the elements having significant z-scores. However, NMF is quite time-consuming and requires bulk memory space. For NMF, it is worth noting that not only it requires non-negative input matrices, but also proper normalization step for these input data sets as they have quite different distributions and variability.

#### iCluster

Like NMF, iCluster (Shen et al., 2009, 2012) assumes a regularized joint latent variable, which is similar to *W* in NMF but without non-negative constraints. *H* is the loading factor (coefficient), the imposed sparsity with different types of penalty functions for various data types. iCluster uses *E* to represent the error/noise term, and the underlying decomposition equation is:

(2) $$\begin{array}{l} X=WH+E \end{array}$$

#### iCluster+

The upgraded iCluster+ expands iCluster by making the assumption of different modeling approaches for the relationships of *X* and *W* within different data platforms. It allows for diverse data types including binary, continuous, categorical, and sequential data with different modeling assumptions including logistic, normal linear, multilogit, and Poisson distributions (Mo et al., 2013). The common latent variable vector *W* represents the underlying driving factors that can be used for disease subtype assignment. Least absolute shrinkage and selection operator (LASSO) penalty is introduced to address the sparsity issue in *H* (Tibshirani, 1994). Since this approach requires high computational complexity, it is necessary to preselect the features critical for clustering results (Wang et al., 2014; Speicher and Pfeifer, 2015). Both iCluster and iCluster+ do not require non-negative input data, unlike NMF.

#### Joint and Individual Variation Explained (JIVE)

Another variation of NMF category is Joint and Individual Variation Explained (JIVE) method. JIVE decomposes the original data of each layer into three parts, including an approximation of joint variation across data types, approximation of specific structured variation for each data type, and residual noise. In other words, JIVE factors the original data input matrix (gene expression etc.) into two lower ranked representative portions *W*^*c*^ (shared factor) and *W*^*s*^ (data-specific factor), dependent on *H*^*c*^ and *H*^*s*^ (Lock et al., 2013). *H* matrix is contributed from one sub-matrix *H*^*c*^ common for all data types, and the other sub-matrix *H*^*s*^ specific to each data type.

(3) $$\begin{array}{l} X=W^{c}H^{c}+W^{s}H^{s}+E \end{array}$$

It should be noted that there can be separate loading factors (*H*^*c*^ and *H*^*s*^) for the shared factor and data-specific factor (*W*^*c*^ and *W*^*s*^). The ranks of the two loading factors can be different. An application of JIVE on gene expression data and microRNA data on Glioblastoma (GBM) samples provided information to better characterize samples into different subtypes and strong clues for associations between each input layer (gene expression and microRNA). Based on PCA for factorization, JIVE suffers from outliers, thus the robustness of JIVE is a major concern. L1 penalties are also placed to reduce the dimensions in JIVE, giving non-zero loadings representing larger and significant contributions to the variation of data.

#### Joint bayes factor

On the other hand, an alternate called Joint Bayes Factor, assumes a common factor loadings *H* for both shared and data-specific factor *W*^*c*^ and *W*^*s*^ (Ray et al., 2014). Like JIVE, the original data input (e.g., gene expression data matrix) is decomposed into shared common factors across data types, data-type specific factors, and residual noise. However, unlike JIVE, which introduces sparsity using L1 penalties, the Joint Bayes Factor model assumes a beta-Bernoulli process for both the common factors and data specific factors (*W*^*c*^ *and W*^*s*^; Griffiths and Ghahramani, 2005; Thibaux and Jordan, 2007). For factor loadings (*H*), the model uses the student-t sparseness-promoting prior, to impose sparsity (Tipping, 2001). As a result, both shared features from each data type and unique features for individual layers can be identified for further analysis. One limitation of Joint Bayes Factor lies within the linear relationship between the latent space and the observational space, and it also assumes very close relationship for different levels of data. Joint analysis of gene expression data with CNV data through this approach identified experimentally validated key drivers, as well as important candidates for further validation for ovarian cancer.

(4) $$\begin{array}{l} X=\left(W^{c}+W^{s}\right)H+E \end{array}$$

### Correlation-based analysis

Canonical correlation analysis (CCA), a traditional method to investigate the relationship between two sets of variables, has been modified and applied to the data integration field. In CCA, two datasets can be decomposed as:

(5) $$\begin{array}{l} X=W_{x}H_{x}+E \end{array}$$

(6) $$\begin{array}{l} Y=W_{y}H_{y}+E \end{array}$$

*H*_*x*_ and *H*_*y*_ stand for loading factors for each data set. CCA aims to find the loading factors ($h_{x}^{i}and\;h_{y}^{i}$ representing the *i*^*th*^ column for loading factors) which maximize the correlation:

(7) $$\begin{array}{l} argmax_{H_{x},H_{y}\;}corr\left(Xh_{x}^{i}\;,\;Yh_{y}^{i}\right) \end{array}$$

Traditional CCA doesn't account for dimension reduction techniques to compute the inverse of a covariance matrix. For the integration purpose, penalization and regularization terms are added cooperatively to create more stable and sparse solutions of loading factors. L1-penalized sCCA (sparse CCA) together with elastic net CCA were proposed to filter the number of variables to make the results more biologically interpretable (Parkhomenko et al., 2009; Witten and Tibshirani, 2009). Recent research on CCA includes consideration of grouped effects of features as structures embedded within the data sets, such as structure-constrained CCA (ssCCA) and CCA-sparse group (Chen et al., 2013; Lin et al., 2013).

Partial least squares (PLS) is focused on maximizing covariance and can potentially avoid the issue of sensitivity to outliers. It projects variables onto a new hyperplane while maximizing the variance to find the fundamental relationship between the two sets of data.

(8) $$\begin{array}{l} X=W_{x}H_{x}+E \end{array}$$

(9) $$\begin{array}{l} Y=W_{y}H_{y}+E \end{array}$$

*H*_*x*_ and *H*_*y*_ stand for loading factors for each data set. The aim of PLS is to find the loading factors which maximize the covariance between *W*_*x*_ and *W*_*y*_:

(10) $$\begin{array}{l} argmax_{H_{x},H_{y}\;}\;cov\left(W_{x}\;,\;W_{y}\right) \end{array}$$

However, in some cases such as in high dimensional biological omics data, it is desired to obtain sparse solutions for better interpretations of the result. More recently, sparse solutions of PLS such as sPLS has been shown to perform equivalently with that of the CCA-elastic net (Lê Cao et al., 2009). Other implementations of PLS with different objective functions and various constraints were also reported. For example, sparse Multi-Block Partial Least Squares (sMBPLS) overcomes the limit of two data block computation through redefining the objective function as a weighted sum of latent variables in different layers (*n* ≥ 2; Li et al., 2012). And Sparse Network regularized Partial Least Square (SNPLS) is specialized in identification of gene expression and drug-response relationship co-modules through incorporating gene interaction network structures (Chen and Zhang, 2016). It showed significantly better performance in accuracy compared to sPLS in simulated data.

### Bayesian methods

Bayesian methods have been applied to data integration for over a decade (Imoto et al., 2004; Zhao et al., 2012). The main advantage of Bayesian methods in data integration is that they can make assumptions not only on different types of data sets with various distributions but also on the correlations among data sets. We briefly overview these methods below:

#### Multiple Dataset Integration (MDI)

It offers to model each data set using the Dirichlet-Multinomial Allocation (DMA) mixture model, thus can explore the shared information through deriving statistical dependencies (Kirk et al., 2012). In this approach, the allocation of genes from one data set has an influence on those in another set. Apart from bi-clustering (clustering two dimensions from the same data set simultaneously), MDI can cluster a single dimension (e.g., genes) across multiple data sets, under the assumption that these genes are measured in all different levels. It can be extended flexibly by allowing variable associations from different groups of genes across data types. This method excels in identifying genes having their protein products in the same complex, apart from the co-regulated genes. Finally, after learning the similarity of clusters in different data sets, MDI obtains a single-dimension cluster among all the input data sets.

Prob_GBM is another probabilistic framework to construct patient similarity network, where patients are represented by nodes and phenotypic similarities among the patients are edges (Cho and Przytycka, 2013). This method uses the genetic phenotype, which is the gene expression data of each patient, to assign corresponding disease subtype. Explanatory features (e.g., CNVs, mutations and miRNA expression) are used to explain phenotypic similarities constructed from gene expression data, among patients. Thus, each disease subtype is modeled by a distribution of these features, and each patient is characterized as the mixture of the genetic characteristic of each subtype. Finally, patients are labeled by the most likely subtype assignment. This method considers the biological relationships among several genomic layers including mutation, CNVs, and miRNA expression data, but it is limited in terms of the types of input data.

#### Patient-Specific Data Fusion (PSDF)

It is based on a two-level hierarchy of Dirichlet Process model, a widely used Bayesian non-parametric model for clustering (Yuan et al., 2011). It checks the concordance between expression and the CNV for each patient. Moreover, it also selects informative features and estimates the number of disease subtypes from the given data. However, this method limits the input for only two types of data (gene expression and CNV), thus reduces its flexibility within multi-platform analysis.

#### Bayesian Consensus Clustering (BCC)

This method is a flexible clustering approach capable of simultaneously modeling the dependence and the heterogeneity of various data sources (Lock and Dunson, 2013). It allows for separate clustering of the objects from each data source and performs *post-hoc* integration of separated clusters. Consensus clustering is applied to model the source-specific structures as well as to determine the overall clustering.

#### COpy Number and EXpression In Cancer (CONEXIC)

It is a Bayesian network-based method to integrate CNV and gene expression data (Akavia et al., 2010). A score-guided search is applied to identify the combination of modulators (genes). A ranked list of high-scoring modulators (candidate driver genes) is produced, representing genes that are both correlated with differential gene expression modules across tumor samples and are present in significantly amplified/deleted regions. The key feature of the CONEXIC goes beyond identifying mutation drivers, as it provides the insights into the roles of drivers and associated genes.

### Network-based methods

Network-based approaches can identify modules, symbolic representations of the disease-associated mechanisms. In this regime, nodes represent genes and edges are links between two genes if there exists interaction between them. Under the unsupervised category, network-based methods are mostly applied for detecting significant genes within pathways, discovering sub-clusters, or finding co-expression network modules (Vaske et al., 2010; Wang et al., 2014; Bonnet et al., 2015).

#### PAthway Representation and Analysis by DIrect Reference on Graphical Models (PARADIGM)

It is a probabilistic graphical model framework to infer patient-specific genetic variations, with the incorporation of curated pathway interactions among genes (Vaske et al., 2010). PARADIGM converts each pathway in National Cancer Institute (NCI) Pathway Interaction Database (PID) into a distinct probabilistic model, represented as a factor graph with both hidden and observed states. Variables in the graph are used to describe molecules, protein-coding genes and complexes (all three assigned as physical entities) apart from gene families and abstract processes. A pathway is modeled as a directed acyclic graph where edges are defined as either positive or negative influence on the downstream nodes, and the nodes are determined by combining all input signals. The output of PARADIGM includes the integrated pathway activity (IPA) score, representing a patient specific measure for the degree of alteration for a specific pathway, through summarizing information from input data sets such as gene expression and CNVs. PARADIGM claims to provide more robust and consistent signatures for subgrouping patients through demonstration in breast cancer and glioblastoma samples. However, in PARADIGM pathways are measured independently, and interactions among pathways are not considered.

#### Similarity Network Fusion (SNF)

This approach aims at discovering the patient subgroup clusters. SNF integrates different data types by constructing a network of samples (rather than genomic features) for each data type, and then fusing these networks into one comprehensive network (Wang et al., 2014). It has two main steps for data integration: First, it constructs a sample-by-sample similarity matrix for each data type, acting as an individual network. Similarity matrices help to identify universal clusters and networks. It also detects different types of data that give support to each connection in the network. Then, by using the non-linear method of message passing theory (KNN and graph diffusion), SNF fuses different similarity matrices and networks, making the combined networks more coherent during each iteration. As a result, weak similarities (e.g., noises) are removed, and strong similarities are added. SNF is relative flexible without constraints for input data format and but only matched samples across different omics layers. By outputting combined similarities among patients across various layers, SNF offers deeper insight into the comprehensive biological relationship, beyond the scope of basic classification and subtyping methods.

#### Lemon-tree

It is another unsupervised method focused on reconstructing module networks (Bonnet et al., 2015). After finding co-expressed clusters from the expression data matrix, Lemon-Tree helps to identify consensus modules and upstream regulatory programs through ensemble methods. First, a gene expression matrix is employed to infer co-expressed gene clusters through a model-based Gibbs sampler. Consensus modules of co-expressed genes are merged through spectral edge clustering algorithm with an ensemble of the gene cluster results. On the other side, additional candidate regulator types of data such as miRNA expression, CNV and methylation data are combined with the consensus module to infer a regulatory score calculated by a decision tree structure. The above separation of module learning and regulator assignment steps provides much more flexibility allowing combination with the other methods. According to the authors, Lemon-Tree has the advantage of inferring more closely related short-path networks with more significant gene ontology-related categories, in comparison to CONEXIC. However, it limits the input data types to be only gene expression and additional one data type, as it is focused on finding co-expressed clusters.

### Multiple kernel learning and multi-step analysis

Multi-step (or multi-stage) methods are commonly used to find relationships between the different data types first, and then between the data types and the trait or phenotypes (Ritchie et al., 2015). Kernel methods are defined by the use of kernel functions, which enables to operate in a high-dimensional feature space by simply computing the inner products among the images of all pairs of data in the feature space (Hofmann et al., 2008). Kernel-based data integration methods are usually multi-steps, thus we exemplify multi-kernel and multi-step methods together.

#### Regularized Multiple Kernel Learning Locality Preserving Projections (rMKL-LPP)

This approach can deal with multiple omics data integration such as gene expression, DNA methylation, and microRNA expression profiles (Speicher and Pfeifer, 2015). It is an extension of the current multiple kernel learning with dimensional reduction (MKL-DR) method, where the data are projected into a lower dimensional and integrative subspace. A regularization term is added to avoid overfitting during the optimization procedure, and it allows using several different kernel types. The Locality Preserving Projections (LPP) is applied to conserve the sum of distances for each sample's k-Nearest Neighbors. The finalized clustering is done through applying k-means on the distance summation. Compared to SNF, rMKL-LPP claims to offer comparable results with much more flexibility, as it provides different choices of dimension reduction methods and a variety of kernels per data type.

#### CNAmet

It is a state-of-the-art multi-step integration tool for CNV, DNA methylation, and gene expression data (Louhimo and Hautaniemi, 2011). The major goal of CNAmet is to identify genes that are both amplified and upregulated or both deleted and downregulated. This tool integrates CNV and DNA methylation data through their functions on gene regulation. The underlying hypothesis is that the gene upregulation is due to both amplified copy number and hypomethylation, whereas gene downregulation is the result of deleted copy number and hypermethylation. It uses three steps to detect the significant genes: weight calculation, score calculation, and significance evaluation. During the first weight calculation step, the signal-to-noise statistics is calculated to measure the copy number and methylation aberrations relative to the expression values. In the second score calculation step, the weight values are combined to infer a deterministic score, which informs the causes of the alterations in the gene expression. Finally, the permutation test is performed on the combined scores and the *P*-values are corrected. Identification of the genes which are synergistically regulated by methylation and CNV data leads to better characterization of these genes and better understanding of biological process underlying cancer progression.

#### In-Trans Process Associated and *Cis*-Correlated (iPAC)

It is a multi-step method to identify genes that are in-*cis* correlated through integrating gene expression and CNV data, as well as genes that are in-*trans* associated to the biological processes (Aure et al., 2013). The novelty of this method is the capability to adjust for confounding effects of co-occurring copy number aberrations. This analysis module combines correlation analysis, regression, gene set enrichment, and adjustment for co-occurring copy number aberrations with avoidance of confounding effects. In the in-*cis* correlation, it proposes a linear model where log gene expression is a linear function of log copy number and noise. In the in-*trans* association, it imposes a direct integration through a statistical enrichment step to get the confidence level of in-*trans* associations between the genes and biological processes.

## Supervised data integration

**Figure 2:**
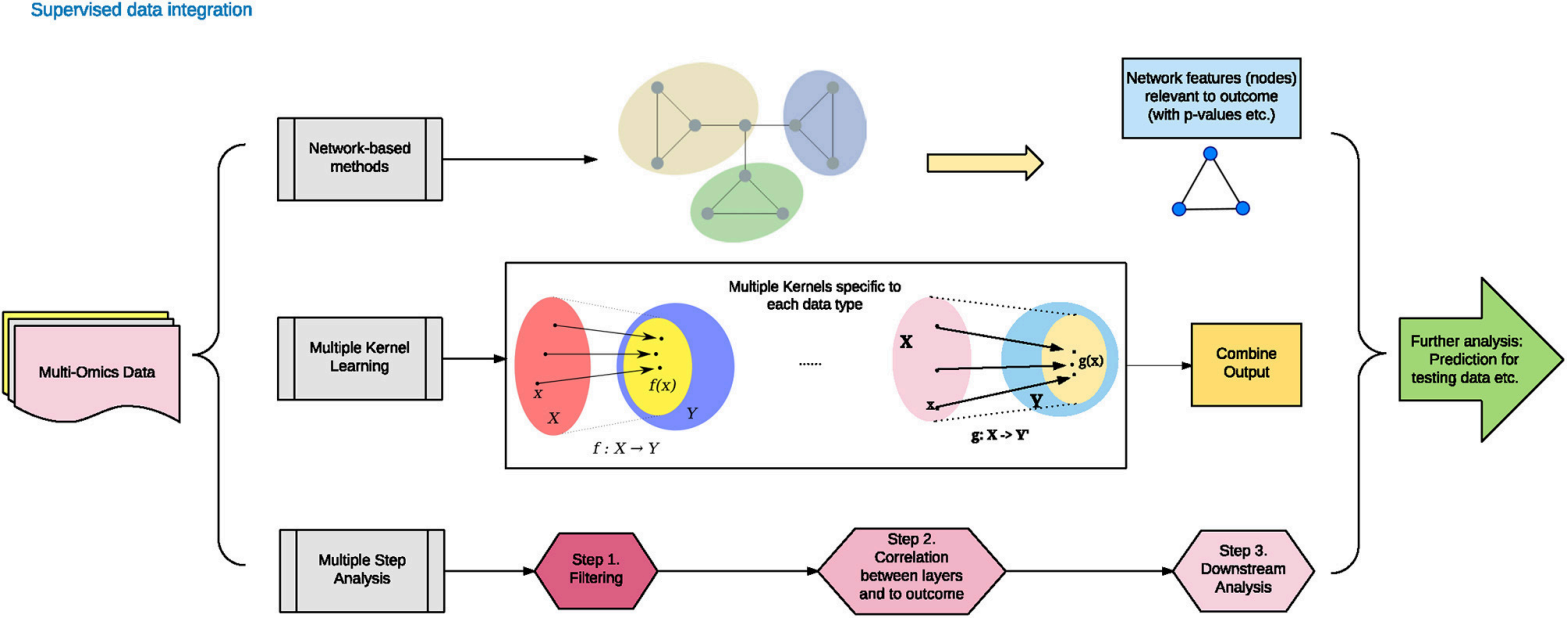
Supervised data integration methodology.

Contrary to the unsupervised data integration methods, the supervised methods consider the phenotype labels of samples (disease or normal), and invoke machine training approaches to evaluate the models. Supervised data integration methods are built via information of available known labels from the training omics data. In the following section, we enlist representative network-based, multi-kernel and multi-step based methods (Figure 2 and Table 1).

### Network-based methods

Analysis Tool for Heritable and Environmental Network Associations (ATHENA) is a neural network approach to integrate different omics data with a supervised model which can further be extended to do prognosis analysis (Kim et al., 2013). In ATHENA, grammatical evolution neural networks (GENN) algorithm is utilized to train individual models from different data platforms. Based on neural networks, grammatical evolution algorithm is utilized to train the model with selected features that are less noisy and significantly associated with clinical outcomes. After selecting the features, individual models are summed up to a final integrative model, which can be utilized for multiple purposes including diagnosis and prognosis. Overall, ATHENA provides a comprehensive way of visualizing genomics data's correlation with clinical features such as survival outcomes, making it stand out compared to other network-based integration methods. One limitation of ATHENA lies in lacking interaction terms among different layers, as the features are selected from individual data type first and then combined into an integrated model.

#### jActiveModules

It is another network-based Cytoscape plug-in which seeks underlying network hotspots through the integration of gene expression, protein-protein interaction, and protein-DNA interaction data (Ideker et al., 2002). This method is based on the hypothesis that molecular interactions linking the genes are more likely to correlate expression profiles than randomly chosen genes in the network. This method requires an external input of significance measurements over genes for significance calculation of sub-networks. The external filtering step is a supervised feature selection for genes based on the *P*-values in the differential expression tests, while the integration method itself doesn't require additional outcomes as inputs. Through random sampling approach and iterative calculations, jActiveModules determines the highest-scoring sub-network circuits in a full network of molecular interactions, leading to further biologically interesting discoveries (Cline et al., 2007). Compared to other clustering methods, jActiveModules is subject to the molecular interaction network and can include genes without dramatically expression fold changes.

Another network-based integration method (Ruffalo et al., 2015) claims to identify key proteins at sample-level using propagated protein networks, based on integrated mutation and differential gene expression (DGE) data sets. Propagated mutation and DGE profiles for each gene are generated with the help of prior knowledge in PPI framework (Schaefer et al., 2012). Feature selection is then done on these propagated profiles in a supervised fashion, with top features being most relevant to outcomes, and a final set of proteins is selected based on the network proximity across the samples. The final step involves logistic regression using the selected genes. This method is useful to find the hidden repertoire of genes/proteins at pathway level with impact on tumor progression/clinical outcome, which might be overlooked by individual mutational or differential expression analysis.

### Multiple kernel learning

#### Semidefinite Programming/Support Vector Machine (SDP/SVM)

It offers a pioneering kernel-based framework for data integration (Lanckriet et al., 2004). Each data set is represented by a specific kernel function that defines similarity between pairs of entities. Then the kernel functions, derived from different omics data, are combined directly using the SDP (Semidefinite Programming) techniques to reduce the integration problem to a convex optimization problem. The SDP method outperforms the classifier trained with a naïve and unweighted combination of kernels. Different kernels correspond to different transformation of the data, with an extraction of a specific type of information from each data set. The Fast Fourier Transform (FFT) kernel is specific for the membrane protein recognition, by directly incorporating information of hydrophobicity patterns. Higher-order polynomials such as radial basis kernels can be used to capture higher-order non-linear associations of a trait with genotypes. Diffusion kernels are applied to exploit unlabeled data. SDP/SVM is a prototype work for kernel-based data integration methods (published in 2004) and doesn't include a programming package.

Feature Selection Multiple Kernel Learning (FSMKL) is another method implementing the multiple kernel learning-based supervised learning (Seoane et al., 2014). This new scheme uses the statistical score for feature selection per data type per pathway. By employing additional kernels based on clinical covariates, it improves the prediction accuracy for cancer detection. Multiple kernel learning constructs classifiers with a decision function dependent on a variety of different types of input data (gene expression & CNV) using pathway-based kernels. Each type of data (omics) is encapsulated into an object called base kernel; a composite kernel is built as a linear combination of these base kernels. To further incorporate biological information into the algorithm, not only individual feature (such as genes) are independently used to construct kernels, but also specific groups of genes, which are known to have membership from a KEGG pathway, are combined to derive other base kernels. The most appropriate decision function over kernels is finalized after feature selection steps, contributing to an integrative function over base kernels. This method stands out among other kernel-based methods with the inclusion of pathway-based information to build kernels, as prior knowledge. Pathway membership is a central criterion for FSMKL to group samples into different clusters, bringing more biological knowledge compared to basic statistical priors from other methods. Combining clinical factors along with high-throughput profiles into the classifier also brings power for prediction accuracy. FSMKL claimed that this method competes with the winner methods from the DREAM challenge for breast cancer prognosis.

### Multi-step analysis

Integrative Bayesian analysis of genomics data (iBAG) is a flexible tool to integrate data from an arbitrary number of platforms (Jennings et al., 2013). A hierarchical model is built to incorporate the information from different genomic layers with biological sense. Basically, this multi-step analysis consists of two-stage models. The first-stage mechanistic model is a regression model which is constructed to partition gene expression data into small segments including methylation principal component, CNV principal component and unknown components other than the previous two. In the second stage of developing clinical a model, clinical data such as binary outcome and survival information is modeled as the response of joint regression from those factors in the previous regression. Normal-Gamma (NG) prior is applied to improve the effect size estimation and address sparsity. This study considers gene expression, methylation and CNV data, in specific, to identify genes having a significant impact on patient survival. The hypothesis of this research lies in the linear relationship between methylation data and CNV, together with the effect of gene expression on survival outcome. These relationships may not reflect the actual biological process underneath, thus the output prognostic genes may not be considered as causal factors. Independent functional experiments and other datasets are needed to validate the results.

#### Multiple Concerted Disruption (MCD)

This method allows to integrate CNV, DNA methylation, and allelic (loss of heterozygosity) status to find genes representing key nodes in the pathways as well as genes which exhibit prognostic significance (Chari et al., 2010). For each differentially expressed gene, the CNV, methylation and allelic statuses are examined for whether the observed expression change would match the expected change in the DNA level. This multi-step tool can be broken down into several sequential steps: First, a set of most frequent differentially expressed genes is identified for each sample with a pre-defined frequency threshold. Next, this subset of genes is further checked according to the concerted pattern of the expression change and also in at least another DNA dimension (CNV, methylation or loss of heterozygosity). Finally, genes are selected which have a role in multiple disruption mechanisms and changes in expression. As a pioneering work in data integration field, MCD offers a biologically sensible way to select genes step wisely by incorporating parallel analysis in genomic and epigenomic layers. However, it is more like a filtering step to finalize a group of genes rather than a systematic way to integrate information embedded from multiple layers.

#### Anduril

It is a bioinformatics workflow proposed to generate integrative results from multiple platforms into a report for biologists for better comprehension (Ovaska et al., 2010). It is a flexible and intuitive analysis tool, which facilitates the integration of various data formats, bio-databases and analysis techniques to identify the genes and loci with high impact on survival. It supports data input including gene expression, miRNA expression, methylation, CNV, exome sequencing, and array CGH data. The workflow maneuvers to manage and automate the sequence of multi-platform analyses from importing the raw data to reporting and visualizing the results. The generated comprehensive website collects all the analyses results and thus facilitates the interpretation of the data. However, this framework is more of a platform to collect and process multiple types of data, rather than a package that performs data integration with sophisticated statistical or machine learning methods.

## Semi-supervised data integration

Semi-supervised integration methods, lies between supervised and unsupervised methods, takes both labeled and unlabeled samples to develop learning algorithm. Most of the semi-supervised data integration methods are graph-based, as illustrated with a few examples below (Table 1).

### GeneticInterPred

It is a tool to predict the genetic interactions through combining the protein-protein interaction, protein complex, and gene expression data (You et al., 2010). This method starts with building a high-coverage, high-precision weighted functional gene network by integrating gene expression, protein complex, and protein-protein interaction data. The topological properties of the protein pairs and gene expression in the function gene network are used as input for the subsequent classification step. A weight matrix is built summarizing the information among the edges in the graph, which is made symmetric. A similarity matrix is inferred from the weight matrix iteratively, using local connectivity in the gene network until convergence. Using connected weighted graph, the graph-based semi-supervised learning (SSL) method can infer the information of the unlabeled interactions in the graph. The final product is a classification matrix where all the unlabeled interactions are assigned. This method is specifically designed for prediction of genetic interaction from integrated functional gene networks. Moreover, the semi-supervised idea of inferring unlabeled data from labeled data in the connected graph of similarity matrix can be applied to clinical predictions like cancer diagnosis and prognosis.

Another pilot framework employing graph-based SSL uses the multi-level genomic data sources (including CNV, gene expression, methylation, and miRNA expression) for molecular classification of clinical outcomes (Kim et al., 2012). This method uses the genomic relationship to define the edges (relationship) between the nodes (samples), and the unlabeled samples are influenced by the propagation of their annotated neighbors. In the end, diverse graphs from different layers are combined by the linear combination of coefficients for the individual graphs. It allows the flexibility to extend to integrate multiple levels of genomic data (*n* > 3), while preserving the level-specific properties from the different and heterogeneous layers. In summary, this work pioneered in combining genomics, epigenomics, and transcriptomics data to predict for cancer phenotypes. However, the interaction relationships among different layers were not considered, such as the regulatory role of methylation or microRNA on gene expression.

## Biological insights from data integration methods

By now we discussed a variety of integration methods in three categories: unsupervised, supervised, and semi-supervised. Unsupervised methods recruit different approaches (factorization, Bayesian, network etc.) to explore their biological profiles to assign objects into different subgroups (clusters). Supervised methods employ the biological information of labeled objects to derive patterns for different phenotypes and assign labels to unlabeled data by comparing the patterns. Semi-supervised methods are mostly building object-wise similarity networks through compiling omics data and assign labels to unknown objects through their relationship to labeled objects.

Interactions among different layers are major concerns for data integration strategies. The corresponding mapping relationship among different layers such as methylation to gene expression, microRNA to gene expression etc. should not only be considered independently but also together during the integrative process. At the initiating stage of data integration, many integrative methods are independently working on different layers (such as multi-step analysis) and then find the common subset of biological identities (e.g., genes) which are significantly differentially expressed in each layer. The more recent emerging state-of-the-art integrative tools are considering interactions while integrating different layers. SNF, for example, tries to integrate patient-wise similarities as a combined network, which both strengthens the coherent relationships from each network and reduces the noise of weak signals from the individual network. iCluster+, on the other hand, aims to discover the common latent variable (structure) from all different omics layers with different modeling assumptions. Thus, the internal relationship of different layers is considered as the driving factor that acts in a concerted manner from each omics data.

## Data integration for survival prediction

Nowadays cancer prognosis prediction is a keen point of interest for physicians, cancer patients, and healthcare-providers. Information about cancer prognosis helps all kinds of decisions regarding the patient management and therapeutic treatments etc. (Hagerty et al., 2005; Rabin et al., 2013). Prognostic biomarkers have been used for more effective selection of patient subgroups with different therapeutic strategies (Huang et al., 2014, 2016). Therefore, molecular data with increasing power to detect personalized molecular characteristics has been studied widely in the past decade (Van 't Veer et al., 2002; Kim and Ritchie, 2014). However, methods to integrate multi-omics data optimized for prognosis prediction (rather than being *post-hoc* evaluation) are far fewer (Table 1). We enlist some representative methods below:

### CoxPath

It is a vector space integration methodology that can handle CNV, gene expression, DNA methylation, and miRNA expression data (Mankoo et al., 2011). First, the Spearman rank correlations among different data types are computed, and separate cut-offs are used to filter the correlated data pairs. After the filtering, L1-penalty is combined with Cox proportional hazards model for feature selection and model shrinkage simultaneously. This metric is a typical multi-step analysis method to predict survival.

### Metadimensional Knowledge-Driven Genomic Interactions (MKGIs)

This framework performs knowledge-based integration of multi-omics genomics data at pathway level (Kim et al., 2014, 2016), to predict the clinical outcome of patients. The strength of the framework lies in capturing genomic interactions by integrating pathways with the metadimensional models to achieve improved prognosis and diagnosis. In transformation phase, each genomic layer is converted to pathway-based knowledge-driven matrix. In modeling phase, an evolutionary algorithm-based method called grammatical evolution neural networks (GENN) is used to develop knowledge-driven models for predicting clinical outcome. GENN is essentially an artificial neural network (ANN) based on grammar rules, which optimizes the high-dimensional input features, network structure, and weights. Further, different genomic interaction models are integrated to develop MKGI models to predict survival, stage and grade. This method concludes that knowledge-driven (pathway-based) genomic models overall perform better than single genomic-based models where gene expression is most contributing at the pathway level.

## Conclusion

A plethora of data is accruing with the high-end experimental set-ups in the field of pathology. Advanced technologies are coupled with the computational challenges to deliver the most relevant biological interpretation of data. In this direction, a considerable number of tools have been developed to make the most out of the multi-tier data sets. This review summarizes the diverse computational tools developed over the years, their advantages and limitations. As this field flourishes, comparisons among different methods will be critical, to aid decision-making by investigators with big data needs. Despite these accomplishments, there needs to be more accurate and efficient tools, especially when clinical outcome (e.g., survival) is to be modeled. Biological knowledge guided integrative methods will continue to be desirable, with consideration of the interactive relationship among different omics layers. Moreover, given that most studies have only a single or a few omics layers, integrating heterogeneous data from multiple cohorts, rather than coupled samples, will need rigorous investigation (Wei et al., 2016). For the purpose of precision medicine, additional benefits may be obtained by integrating omics data with other data types, such as imaging and electronic health record (EHR) data.

## Author contributions

LG envisioned the project, SH and LG designed the workflow. SH, KC, and LG wrote the manuscript. All authors have read, revised, and approved the final manuscript.

### Conflict of interest statement

The authors declare that the research was conducted in the absence of any commercial or financial relationships that could be construed as a potential conflict of interest. The reviewer JC and handling Editor declared their shared affiliation, and the handling Editor states that the process nevertheless met the standards of a fair and objective review.
